# Members of the *Lactobacillus* Genus Complex (LGC) as Opportunistic Pathogens: A Review

**DOI:** 10.3390/microorganisms7050126

**Published:** 2019-05-10

**Authors:** Franca Rossi, Carmela Amadoro, Giampaolo Colavita

**Affiliations:** 1 Diagnostica Specialistica, Sezione di Isernia, Istituto Zooprofilattico Sperimentale dell’Abruzzo e del Molise “G. Caporale”, C.da Breccelle Snc, 86170 Isernia, Italy; 2 Medicine and Health Science Department “V. Tiberio”, University of Molise, Via de Santis, 86100 Campobasso, Italy; carmela.amadoro@unimol.it (C.A.); colavita@unimol.it (G.C.)

**Keywords:** *Lactobacillus* species, opportunistic pathogens, infections, risk factors, predisposing factors, virulence

## Abstract

Microorganisms belonging to the *Lactobacillus* genus complex (LGC) are naturally associated or deliberately added to fermented food products and are widely used as probiotic food supplements. Moreover, these bacteria normally colonize the mouth, gastrointestinal (GI) tract, and female genitourinary tract of humans. They exert multiple beneficial effects and are regarded as safe microorganisms. However, infections caused by lactobacilli, mainly endocarditis, bacteremia, and pleuropneumonia, occasionally occur. The relevance of *Lactobacillus* spp. and other members of the LGC as opportunistic pathogens in humans and related risk factors and predisposing conditions are illustrated in this review article with more emphasis on the species *L. rhamnosus* that has been more often involved in infection cases. The methods used to identify this species in clinical samples, to distinguish strains and to evaluate traits that can be associated to pathogenicity, as well as future perspectives for improving the identification of potentially pathogenic strains, are outlined.

## 1. Introduction

Bacteria currently classified in the genus *Lactobacillus* are a paraphyletic group of gram-positive, non-spore forming, mostly non-respiratory, but aerotolerant, lactic acid bacteria (LAB), comprising at this time more than 237 species and 29 subspecies (http://www.bacterio.net/lactobacillus.html) [1]. Morphologically, they can be elongated or short non-motile rods, frequently found in chains and sometimes bent. They produce lactic acid as a major end-product of carbohydrate fermentation.

Lactobacilli are part of the normal human microbiota that colonizes the mouth, gastrointestinal (GI) tract, and female genitourinary tract. Moreover, these bacteria have been used for centuries for food and feed fermentation processes aimed at the transformation of perishable raw materials of animal or plant origin into more preservable products. Their activities are relevant to the production of dairy products, bread, sausages, fermented vegetables, wine, and silage.

According to the type of sugar fermentation pathway, lactobacilli fall into the following three groups, all including species that are industrially exploited: (i) obligately homofermentative, that produce only lactic acid as an end product of carbohydrate metabolism through the glycolysis pathway; (ii) facultatively heterofermentative, that produce a mixture of lactic and acetic acid as end products of carbohydrate metabolism through the glycolysis or the phosphoketolase pathway, and; (iii) obligately heterofermentative, that produce lactic and acetic acid, or ethanol, and CO_2_ as end products of carbohydrate metabolism through the phosphoketolase pathway [2].

The genome size of *Lactobacillus* spp. is highly variable, ranging between about 1 and more than 4 Mb. Genome size also varies within a single species [3] as a result of genome decay in strains adapted to specialized niches where genes encoding for multiple substrate utilization are lost [4].

Based on whole genome phylogeny, genera *Fructobacillus*, *Leuconostoc*, *Oenococcus*, *Pediococcus*, and *Weissella* were found to descend from the most recent common ancestor of *Lactobacillus*, so that they constitute internal branches of the *Lactobacillus* taxon for which the designation "*Lactobacillus* genus complex" (LGC) has been proposed [5]. For this reason, members of the LGC not classified as *Lactobacillus* spp. were also considered in this review.

Zheng et al. (2015) [3] found a good correspondence between metabolic groups and phylogenomics based on 172 concatenated protein sequences encoded by single copy genes of core genomes and key enzymes of metabolic pathways.

LGC organisms better characterized physiologically and technologically are those of highest relevance for natural or industrial food fermentation, probiotic properties, and biotechnological applications. In Table 1, those most frequently used in food technology and as probiotics are listed, together with type of metabolism, main ecological niche, and technological applications.

Culture-independent DNA-sequence analysis put in evidence that autochthonous *Lactobacillus* organisms represent, at most, 1% of the total bacterial population in the distal human gut. Their number changes in some diseases, such as Crohn’s disease, human immunodeficiency virus (HIV) infection, rheumatoid arthritis, multiple sclerosis, obesity, type 1 and 2 diabetes, irritable bowel syndrome, and prenatal stress. However, the role of autochthonous intestinal lactobacilli in disease prevention and treatment must be still elucidated [24].

A metagenomic analysis on a human subject showed that over a period of two years, more than 50 *Lactobacillus* species, and individual *Lactobacillus* genotypes, were repeatedly detected in numbers of up to 10^8^ cells/g in the stool [25], suggesting that a persistent population of lactobacilli could inhabit the gastrointestinal tract (GIT) of individuals.

*Lactobacillus* species inhabiting human GIT and isolated from faeces comprise most of the microorganisms listed in Table 1 [23,26]. The species *L. antri*, *L. gastricus*, *L. kalixensis*, *L. reuteri*, and *L. ultunensis* have been isolated from the stomach mucosa [27]. Lactobacilli also occur naturally in the human mouth [28]. Another site colonized by lactobacilli is the vagina, where *L. crispatus*, *L. gasseri*, *L. jensenii*, *L. vaginalis*, and *L. iners* are commonly found [29].

The efficacy of lactobacilli as probiotics derives from their ability to tolerate very low pH values, which allows them to survive transit through the stomach, and adhere to the mucus layer by surface structures, such as pili and cell-wall anchored proteins [30]. Some of their beneficial activities are favoring GIT health by inhibiting the growth of pathogenic organisms with the production of lactic acid and other metabolites. Some *Lactobacillus* strains are able to immunomodulate human cells and elicit an anti-inflammatory response [31]. In addition, some strains produce antioxidants [32].

As other probiotics, they are sold as constituents of food, food additives, or food supplements, but control on their use to safeguard consumer’s health needs to be improved [33].

*Lactobacillus* organisms are rarely associated with pathology in immunocompetent people, but in the presence of risk factors and underlying conditions, they can cause infections such as endocarditis, bacteremia, neonatal meningitis, dental caries, and intra-abdominal abscesses including liver abscess, pancreatic necrosis infection, pulmonary infections, pyelonephritis, meningitis, postpartum endometritis, and chorioamnionitis [34,35].

In a retrospective analysis carried out in Argentina between January 2012 and July 2017, *Lactobacillus* spp. were isolated from patients with bacteremia (67%), meningitis, empyema, urinary infection, vaginosis, and hepatic abscess and underlying conditions such as cancer, surgery interventions, diabetes, and intestinal malformation. However, it is not clear from the report if it was ascertained that these organisms were the primary cause of infection. *L. rhamnosus* was most commonly isolated, followed by *L. fermentum*, *L. paracasei*, *L. oris*, *L. gasseri*, *L. iners*, and *L. salivarius* [36].

A recent systematic review of case reports and case series of infection complications after probiotic treatments found that both *Lactobacillus* spp. and *Pediococcus* spp. were involved as causative agents, among other probiotic organisms of common use [37].

Finally, members of the genus *Weissella*, that can be isolated from a variety of habitats including raw milk, feces, saliva, breast milk, urine, and fermented food, have been implicated in cases of bacteremia, abscesses, prosthetic joint infections, and infective endocarditis as a possible consequence of translocation after disruption of the mucosal barrier caused by surgery or therapies [38].

This review presents the evidences for the behavior of LGC organisms as opportunistic pathogens able to cause different types of infection and the related risk factors and predisposing health conditions or medical treatments. The scope is increasing awareness that, for immunocompromised individuals, or those affected by particular medical conditions, these bacteria can represent a hazard and must be used more cautiously as health promoting microorganisms than it is currently done.

## 2. Members of LGC as Opportunistic Pathogens

The risk factors most commonly reported for *Lactobacillus* infections are diabetes mellitus, pre-existing structural heart disease (in infective endocarditis cases), cancer (especially leukemia), total parenteral nutrition, broad spectrum antibiotic therapy [39,40], chronic kidney disease, inflammatory bowel disease, pancreatitis [41], chemotherapy, neutropenia, organ transplantation (especially liver transplantation) [42], HIV infection [43], and steroid use [44].

Moreover, perinatal infections caused by lactobacilli indicate preterm neonates as a population category at risk. Though a meta-analysis indicated that probiotics reduce the incidence of necrotising enterocolitis and all-cause mortality in preterm infants, excluding infants with a birth weight of <1000 g, cases of infections in premature infants have been reported. These include late-onset sepsis due to *L. rhamnosus* following a laparotomy, amnionitis, and neonatal meningitis, cases of bacteremia, lactobacillemia of amniotic fluid origin, *L. rhamnosus* GG bacteremia associated with probiotic use in a child with short gut syndrome, and *L. rhamnosus* infection in a child following bone marrow transplantation [45,46,47].

Experiments with athymic mice have shown the potential for probiotics to cause sepsis in immune deficient neonates. This possibility was supported by case reports of probiotic sepsis in humans [48].

The most common predisposing events for *Lactobacillus* infections are dental manipulation, poor dental hygiene, intravenous drug abuse, abdominal surgery, colonoscopy, probiotic use, and heavy dairy product consumption [49].

Recent opinion articles invite safety assessments to be conducted for *Lactobacillus* probiotics, since they represent a risk for individuals with underlying medical conditions [33,50]. In particular, Cohen (2019) [33] stated that the ability of these strains to infect humans is not controversial and that live bacteria sold as commercial probiotics are capable of infecting immunocompromised hosts and have well-established “inherent infective qualities”.

Theoretically, the potential pathogenicity of probiotics may be enhanced in strains selected on the basis of the capacity to adhere to the intestinal mucosa, a trait that is considered important for their mechanism of action. Indeed, adherence can favor translocation across the intestinal barrier and ability to cause infections. The finding that *Lactobacillus* spp. isolated from blood adhere to intestinal mucus in greater numbers than isolates from human feces or dairy products supports the relationship between mucosal adhesion and pathogenicity [34].

### 2.1. Infections Caused by Members of the LGC

#### 2.1.1. Endocarditis

Among infections caused by lactobacilli, endocarditis, with or without bacteremia, is the most common. It occurred in patients who had dental extractions or gingival bleeding after toothbrushing [51,52], suggesting that these could be considered risk factors, especially in the presence of underlying immunosuppression and valvular heart disease [53].

An *L. rhamnosus* endocarditis case was reported in an 80 year old man who frequently consumed yogurt containing the organism following an upper endoscopy. This patient required aortic and mitral valve replacement for a cure. Cases of *Lactobacillus* endocarditis have also been described following colonoscopy [54]. Patients with hereditary hemorrhagic telangiectasia (HHT) are also exposed to this infection because of telangiectasias and arteriovenous malformations (AVMs). In a habitual consumer of fermented dairy products with this pathological condition, the portal of entry was intestine following a colonoscopy [55].

In a middle-aged man, *L. acidophilus* endocarditis led to an aneurysmal rupture of the sinus of Valsalva into the right ventricular outflow tract with fistula formation from the right coronary sinus to the right ventricular outflow tract that required surgical repair with an aortic valve replacement [56]. A case of mitral valve endocarditis due to *Lactobacillus* was recently reported in an 81 year old woman [57].

*P. pentosaceus* caused endocarditis in a 66 year old male in association with *Lactococcus lactis* subsp. *lactis* [58].

The species *L. rhamnosus* and *L. casei* have been most frequently involved in endocarditis, presumably for their ability to induce platelet aggregation and generate fibrin by producing a factor Xa-like enzyme that catalyzes steps of the coagulation process favoring clot formation. It is supposed that these bacteria colonize thrombotic vegetations where they grow, evading host defenses [59].

#### 2.1.2. Bacteremia

*Lactobacillus* bacteremia has been associated with the consumption of probiotics in special medical conditions, including hematopoietic stem cell transplantation [60] and HIV-infection [61].

Bacteremia caused by *Veillonella* and *Lactobacillus* spp., secondary to occult dentoalveolar abscess, was reported in a pediatric patient [62].

In a patient with chronic lymphocytic leukemia and recurrent bacteremia caused by *L. casei/paracasei* and *L. rhamnosus*, the source of infection was unknown, since probiotics had not been assumed and entry from dental infections or the gastrointestinal and urinary tract was excluded [63].

Bacteremia caused by isolates indistinguishable from the *L. rhamnosus* probiotic strain GG based on pulsed field gel electrophoresis (PFGE) typing was associated with a higher mortality rate than bacteremia caused by other *Lactobacillus* species [40].

*Lactobacillus* sepsis was normally resolved with antimicrobial therapy, but in some cases, patients developed septic shock. In other cases, the outcome has been fatal, but due mostly to underlying diseases rather than probiotic sepsis. On the basis of the characteristics of the cases reported, a list of major and minor risk factors for probiotic sepsis was proposed and caution in using probiotics in the presence of a single major risk factor or more than one minor risk factor was suggested. Major risk factors are being immune-compromised and preterm births, while minor risk factors are presence of central venous catheters (CVCs), impaired intestinal epithelial barrier caused by intestinal infections or inflammation, administration of probiotic by jejunostomy, concomitant administration of antibiotics to which the probiotic is resistant, probiotics with properties of high mucosal adhesion or known pathogenicity, and cardiac valvular disease (*Lactobacillus* probiotics only) [34].

#### 2.1.3. Pleuropneumonia

*Lactobacillus* species were a primary cause of pleuropneumonia without bacteremia, especially in immunocompromised patients. From 1982 to 2016, 15 cases of pleuropneumonia caused by *Lactobacillus* spp. were reported, and involved *L. rhamnosus*, *L. fermentum*, *L. acidophilus*, *L. paracasei*, and *L. coryneformis*. All the patients had severe associated co-morbidities comprising immunosuppression, caused in most cases by AIDS, carcinoma, chronic diseases, and neutropenia. One patient had *Lactobacillus* pneumonia linked to consumption of a probiotic supplement. The route of entry was probably GIT in some patients, the transplanted lung in one patient, ventilator in an immunocompetent patient with thoracic trauma. In one patient, diagnosed with trachea-esophageal fistula, the route of *Lactobacillus* pneumonia was aspiration of a probiotic strain. Only one patient had concurrent lactobacillemia. The authors of the study suspected that infections due to *Lactobacillus* species are under-reported because appropriate growth conditions, such as microaerophily or anaerobiosis, are not applied in clinical microbiology laboratories for their isolation [64].

#### 2.1.4. Meningitis

The first reported case of meningitis in which *Lactobacillus* was isolated from blood and cerebrospinal fluid was in an early-term neonate (38 weeks gestation) within the first day of life. Transmission from the mother’s genital tract to the neonate’s oral mucosa at the time of delivery was identified as the probable route of infection, since no immunological abnormalities, structural defects, or peripartum complications were observed. Another case involved a 10 year old neutropenic child affected by acute leukemia with four successive episodes of *L. rhamnosus* bacteriemia and unknown origin of infection.

A lethal case of meningitis due to *L. rhamnosus* was reported in an 80 year old woman not immunocompromised but with a fistula between the esophagus and the meningeal space, caused by dislodged and eroded plates and screws used several years earlier for cervical spine surgery, that facilitated bacterial translocation.

Meningoencephalitis caused by *L. plantarum* was reported in a 63 year old man with metastatic planoepithelial lung cancer.

Bacteremia and endocarditis, which are the two main manifestations of *Lactobacillus* infection, can lead to the onset of neurological sequelae through mechanisms mediated by embolic material.

This was not the case of the latter patient, who had no signs of endocarditis. Therefore, direct bacterial dissemination from the gastrointestinal tract was hypothesized [65].

#### 2.1.5. Urinary Tract Infections

Cases of urinary tract infections caused by lactobacilli in women have been reported, with symptoms such as chronic pyuria and pyelonephritis with bacteremia, in which *L. delbrueckii* or *L. jensenii* were the causative microorganisms [66,67,68]. A case of urinary tract infection caused by *Lactobacillus* spp. was reported in a newborn [69].

## 3. Virulence of LGC Members

Studies on *Lactobacillus* virulence have regarded mainly the species *L. rhamnosus* and *L. paracasei*, that comprise the most widely used *Lactobacillus* probiotics. These possess potential virulence factors such as production of enzymes which break down human glycoproteins, and proteins that bind extracellular proteins such as fibronectin, fibrinogen, and collagen, which may be important in early stage colonization and adherence. Moreover, some strains have the ability to aggregate human platelets, a trait that has a role in the pathogenesis of various infections [70,71]. The ability to bind fibrinogen is known to help gram-positive pathogens in escaping the immune system and can be sufficient to induce platelet aggregation and lead to infections, such as endocarditis [72]. Recently, a *L. salivarius* isolated from a case of sepsis was found to aggregate human platelets by binding human fibrinogen through a newly described fibrinogen-binding protein [73]. In some species of the genus *Weissella*, genome analysis revealed the presence of potential virulence determinants, such as collagen, adhesins, and hemolysins [74].

Virulence aspects were better studied in the species *L. rhamnosus* that comprises highly effective probiotic strains of wide use.

### 3.1. Focus on L. rhamnosus Pathogenic Potential

#### 3.1.1. Relevance of *L. rhamnosus* as A Probiotic

The species *L. rhamnosus* comprises strains able to exert many proven beneficial effects on health, with *L. rhamnosus* GG as the best studied and most recommended probiotic for the prevention and treatment of conditions like antibiotic associated diarrhea (AAD) caused by *Clostridium difficile*, Crohn’s disease, atopic dermatitis [75,76], and pathological states of the respiratory tract and the vaginal tract [77]. Its use in pediatric patients is justified by its ability to survive to amoxicillin-clavulanate treatment, with relevance for the frequent use of this antibiotic treatment in children [78].

*L. rhamnosus* strain GG has been applied successfully to treat infections caused by vancomycin-resistant *Enterococcus faecium* (VRE). A mechanism explaining the efficacy of this probiotic against VRE intestinal colonization is the prevention of their binding to mucus by competition exerted by the SpaC pilus protein of *L. rhamnosus*, very similar to its counterpart in the clinical *E. faecium* strain E1165 [79].

*L. rhamnosus* GG inhibits biofilm formation by various pathogens, including *Salmonella* spp. and uropathogenic *E. coli*, by the production of lectin-like proteins Llp1 and Llp2. These proteins are also involved in the adhesion capacity of *L. rhamnosus* GG to gastrointestinal and vaginal epithelial cells and could improve the prophylaxis of urogenital and gastrointestinal infections [80].

*L. rhamnosus* strains are endowed with a catalase gene, and are therefore more resistant to oxidative stress, with possible anti-oxidant applications that were recently described [81].

Beneficial effects of *L. rhamnosus* strains proven in vivo in human trials are synthetized in Table 2.

#### 3.1.2. Implication of *L. rhamnosus* in Infection Cases

*L. rhamnosus* has caused infections more frequently than other *Lactobacillus* species. It was implicated in 68 of 85 cases examined, among which 22 were attributable to *L. rhamnosus* GG [92]. Among 60 strains of *Lactobacillus* spp. from blood cultures identified in a retrospective study, *L. rhamnosus* was the most commonly isolated species and was found in blood cultures from 16 patients. Of patients with *L. rhamnosus* bacteremia, 66% were immunosuppressed and 83% had catheters [49]. A case of bacteremia caused by *L. rhamnosus* GG in an adult patient affected by severe active ulcerative colitis under treatment with corticosteroids and mesalazine was associated with candidemia and occurred while the patient was receiving a probiotic formulation containing the same strain (as determined by PFGE typing), and was concomitantly treated with vancomycin, to which the *Lactobacillus* strain was intrinsically resistant [93]. *L. rhamnosus* GG bacteremia was apparently a consequence of the translocation of bacteria from the intestinal lumen to the blood in an immunocompetent 58 year old male suffering from ischemic colitis. The authors of the study underlined that the *Lactobacillus* infection can represent a clue for a serious underlying pathological state [94].

Probiotics are commonly administered to infants to prevent adverse effects of antibiotic treatment and necrotizing enterocolitis. However, the supplementation with *L. rhamnosus* GG has been associated with the development of sepsis with a cause–effect relationship in eight newborns and children. Therefore, physicians must be made aware that supplementation with *L. rhamnosus* GG can cause sepsis in high-risk patients on rare occasions [95].

Other infections caused by *L. rhamnosus* GG were empyema in a human HIV-infected lung transplant recipient receiving a probiotic containing this strain [96], aspiration pneumonia in an eleven month old child with trisomy 21 affected by respiratory syncytial virus (RSV) bronchiolitis who had assumed a probiotic culture containing *L. rhamnosus* GG for 3 months prior to her illness [97], disseminated infection in a 6 day old newborn with intrauterine growth restriction to whom *L. rhamnosus* GG was administered to prevent gastrointestinal complications [98], septic shock caused by yogurt derived *L. rhamnosus* GG in a 54 year old male patient with acute promyelocytic leukemia in second complete remission, and who received high doses of chemotherapy and autologous peripheral blood stem cell transplantation [99], and endocarditis in a patient who regularly ate a yogurt brand labeled as containing *Lactobacillus bulgaricus*, *Lactobacillus acidophilus*, and *L. casei*. In the latter case, though not declared in the label, a *L. rhamnosus* strain identical to blood *L. rhamnosus* isolates based on PFGE and with 2-band difference with the valve isolate was isolated from the product [54].

#### 3.1.3. Methodologies Used for *L. rhamnosus* Identification and Strain Discrimination

Correct species identification and strain discrimination is of utmost importance for the recognition of infection etiological agents. In the case of *L. rhamnosus*, species identification can be carried out by species–specific PCR as described by Alander et al. (1999) [100] or MALDI-TOF MS [97].

Identification can be accomplished by 16S rRNA or *tuf* gene sequencing [49,98].

PFGE with four restriction enzymes, NotI, SfiI, AscI, and FseI, used separately, is the gold standard typing technique applied for the comparison of clinical and probiotic *L. rhamnosus* strains [101].

Another typing method adopted for *L. rhamnosus* strain distinction is repetitive-sequence PCR (rep-PCR) with the primer RW3A. PCR products can be resolved on the Agilent 2100 Bioanalyzer (Agilent, Santa Clara, CA), and the relatedness of the strains can be evaluated using the Diversilab software (bioMérieux, Durham, NC). Identical strains have a similarity index of >99% [97].

Moreover, amplified fragment length polymorphism (AFLP) can be applied as genetic fingerprinting method for *L. rhamnosus* strain distinction [102].

Finally, methods of whole genome comparison have been applied in different occasions for this bacterial species [71,81,103].

#### 3.1.4. Recent Advances in the Study of *L. rhamnosus* Capacity to Behave as Opportunistic Pathogen

Recent developments in the study of *L. rhamnosus* pathogenic potential consist of the analysis of virulence characters at phenotypic and genotypic level.

Comparison of isolates from dental pulp infection with *L. rhamnosus* GG indicated as possible biomarkers for pathogenicity the presence of a modified exopolysaccharide cluster, altered transcriptional regulators of families RpoN, NtrC, MutR, ArsR, and zinc-binding Cro/CI, and changes in the two-component sensor kinase response regulator and ABC transporters for ferric iron. Clinical strains appeared to be segregated on the basis of genomic distance analysis and SNP divergence from *L. rhamnosus* GG and were found to possess only the SpaFED pilus gene cluster instead of SpaCBA and SpaFED, as in the latter strain [103].

Nissilä et al. (2017) [71] studied virulence related characters, i.e. surface exposed structures, complement evasion, platelet aggregation, and biofilm formation in 4 newly sequenced and 12 already described *L. rhamnosus* strains from blood cultures collected from bacteremic patients between 2005 and 2011.

*L. rhamnosus* isolates were clearly different from *L. rhamnosus* GG and from each other at sequence level. The blood isolates showed no common phenotypic trait possibly involved in the persistence in the host, like biofilm formation, platelet aggregation, and pilus production.

Two strain clusters were defined: cluster A, with sequence similarity at nucleic acid level to *L. rhamnosus* GG between 99.942 and 99.984%, and cluster B, with a similarity to *L. rhamnosus* GG between 97.0 and 98.5%. All strains that were found to contain plasmids fell in the genome cluster B. All strains possessed a unique set of LPXTG proteins that are recognized by sortases and are involved in interactions with the environment and *in vivo*.

All the *L. rhamnosus* strains were able to activate the complement system, measured as C3a and terminal pathway complement complex (TCC) formation in serum. However, the strains expressing pili showed a borderline increase in TCC formation compared to the group without pili.

None of the strains bound complement inhibitors C4bp or FH, indicating that *L. rhamnosus*, differently from some pathogens, have not the ability to escape the complement system. Four of the sixteen strains induced platelet aggregation and four strains in cluster B formed stronger biofilm. One strain had both characteristics. Most of these strains belonged to cluster B. There was a significant association between biofilm formation and the presence of the SpaCBA pilus. Similar features are not found in *L. rhamnosus* GG and were observed in pathogenic strains, as reported in earlier studies with strains isolated from infectious endocarditis (5/5 tested strains), laboratory strains (8/16 strains), and strains from infection of aortic aneurysm graft and carcinoma with liver metastasis [70].

Distinctive characters of cluster B compared to strains in cluster A, similar to *L. rhamnosus* GG also in exopolysaccharide (EPS) gene cluster composition, were the presence of only some of the genes in one EPS gene cluster and a different type EPS/CPS cluster comprising 19 genes. This could influence tissue adherence capacity, biofilm formation, and evasion of host defense.

It was concluded that *L. rhamnosus* strains isolated from blood cultures are distinct from *L. rhamnosus* GG, suggesting that use of this probiotic is safe in healthy subjects with a functional immune system.

On the other hand, *in silico* analysis of regulatory motifs in *L. rhamnosus* GG has indicated that some sortases, as well as a fibronectin binding protein, could be upregulated during exposure to stress factors that induce the heat shock response (HSR) [104]. This suggests that the expression of those characters in stress conditions could influence the ability of *L. rhamnosus* GG to be virulent and should be experimentally investigated.

Though the studies on genetic determinants of the capacity to express pathogenicity of lactobacilli or other LGC genera are still limited, these can be synthetized to date as in Table 3 according to the observations in *L. rhamnosus*.

## 4. Conclusions

The capacity of *L. rhamnosus* and lactobacilli in general to behave as opportunistic pathogens has been linked to characters such as platelet aggregation capacity and biofilm formation. Still, little is known on the cell wall structures involved in these activities, so this aspect should be investigated by correlating the cell surface protein profile, including the sortase-recognized LPXTG proteins, with the virulence phenotype.

Moreover, the expression of structures and proteins involved in adherence in different growth conditions should be investigated.

EPS production, which influences biofilm structure and strength, is highly variable among strains and even genetically unstable, being determined by genome regions prone to rearrangements and loss. The implication of type of EPS and production conditions in virulence needs to be better defined by elucidating the link between presence and expression of specific genes and biofilm formation and tenacity on materials used for CVCs or prosthetic heart valve manufacturing.

A better definition of the relationships between expression of specific characters and virulence could lead to the selection of *Lactobacillus* probiotic strains with no intrinsic capacity to pose health risks.

On the other hand, it was shown that belonging to a specific intra-species cluster of plasmid endowed strains from bacteremic patients is per se an indication of potential pathogenicity, so that genome regions specific for those strains could be used to design PCR tests that enable to exclude the membership of probiotic candidates to those clusters.

Since infections due to *Lactobacillus* species are probably under-reported because appropriate growth conditions, such as microaerophily or anaerobiosis, are not applied in clinical microbiology laboratories for their isolation, improved isolation methods should be implemented to correctly estimate the involvement of lactobacilli in infection cases.

## Figures and Tables

**Table 1:** *Lactobacillus* species most frequently used in food technology and as probiotics, type of metabolism, technological applications, and typical ecological niches.

Species	Metabolism	Main Ecological Niches	Main Technological Applications
*L. acidophilus*	homofermentative	GIT, dairy products [6]	Probiotic [6]
*L. brevis*	heterofermentative	Fermented vegetables, GIT [7]	Sourdough fermentation [8]
*L. buchneri*	heterofermentative	Fermented vegetables, dairy products, GIT [9]	Silage fermentation [10]
*L. casei/paracasei*	facultatively heterofermentative	Dairy products, GIT [11]	Cheese production, probiotic [12]
*L. delbrueckii* subsp. *bulgaricus* and *lactis*	homofermentative	Dairy products [13]	Fermented milk and cheese production [13]
*L. helveticus*	homofermentative	Dairy products [4]	Cheese production [4]
*L. plantarum*	facultatively heterofermentative	Fermented food and feed, GIT [14]	Cheese, sausage, fermentation of vegetables, silage production, probiotic [14]
*L. reuteri*	heterofermentative	GIT, skin and mucosae [15]	Probiotic [15]
*L. rhamnosus*	facultatively heterofermentative	Dairy products, GIT [11]	Probiotic [16]
*L. sakei*	facultatively heterofermentative	Meat, vegetables [17,18]	Sausage fermentation [18]
*L. sanfranciscensis*	heterofermentative	Sourdough [19]	Sourdough fermentation [19]
*L. salivarius*	homofermentative	Human and animal GIT [20]	Probiotic [20]
*Oenococcus oeni*	heterofermentative	Grape berries [21]	Wine malolactic fermentation [21]
*Pediococcus acidilactici*	homofermentative	Plant materials, cheese, fermented meat products, GIT [22]	Sausage fermentation, probiotic [22]
*P. pentosaceus*	homofermentative	Plant materials, cheese, fermented meat products, GIT [22,23]	Sausage fermentation, probiotic [22]

GIT: gastrointestinal tract.

**Table 2:** Beneficial effects exerted in vivo by *L. rhamnosus* strains in human trials.

L. rhamnosus Strain	In Vivo Effect
GG	Decrease of total and LDL cholesterol and increase in natural killer activity in elderly persons [82]
	prevention and relief of various types of diarrhea, and treatment of relapsing Clostridium difficile colitis [83,84]
	Anti-inflammatory effect by interleukin-10 generation in atopic children and alleviation of atopic eczema-dermatitis symptoms [85,86]
	reduced duration of respiratory tract infections in in children [87]
SD11	Decrease of oral mutans streptococci [88]
PL60	Production of c9,t11- and t10,c12-conjugated linoleic acids with anticarcinogenic and antiatherogenic activities, reduction of the catabolic effects of immune stimulation, and reduction of body fat [89]
Not specified	modulation of dendritic cells function to induce a novel form of T cell hyporesponsiveness; this mechanism might be an explanation for the observed beneficial effects of probiotic treatment in clinical disease [90]
HN001	increased tumoricidal activity of circulating natural killer (NK) cells significantly correlated with age [91]

**Table 3:** Suggested genetic markers of *L. rhamnosus* pathogenicity.

Infection Caused	Possible Pathogenicity Genetic Marker
Dental pulp infection [103]	a modified exopolysaccharide cluster
	altered transcriptional regulators of families RpoN, NtrC, MutR, ArsR and zinc-binding Cro/CI
	altered response regulator and ABC transporters for ferric iron
Bacteremia [71]	plasmids
	expression of pili
	modification of one EPS gene cluster and a different type EPS/CPS cluster comprising 19 genes

## References

[1] LPSN List of Prokaryotic Names with Standing in Nomenclature. http://www.bacterio.net/lactobacillus.html.

[2] Kandler O. (1983). Carbohydrate metabolism in lactic acid bacteria. Antonie Van Leeuwenhoek.

[3] Zheng J., Ruan L., Sun M., Gänzle M. (2015). A genomic view of lactobacilli and pediococci demonstrates that phylogeny matches ecology and physiology. Appl. Environ. Microbiol..

[4] Papizadeh M., Rohani M., Nahrevanian H., Javadi A., Pourshafie M.R. (2017). Probiotic characters of *Bifidobacterium* and *Lactobacillus* are a result of the ongoing gene acquisition and genome minimization evolutionary trends. Microb. Pathog..

[5] Wittouck S., Wuyts S., Meehan C.J., van Noort V., Lebeer S. (2019). A genome-based species taxonomy of the *Lactobacillus* Genus Complex. BioRxiv Prepr. Serv. Biol..

[6] Bull M.J., Jolley K.A., Bray J.E., Aerts M., Vandamme P., Maiden M.C., Marchesi J.R., Mahenthiralingam E. (2014). The domestication of the probiotic bacterium *Lactobacillus acidophilus*. Sci. Rep..

[7] Fraunhofer M.E., Geißler A.J., Behr J., Vogel R.F. (2019). Comparative genomics of *Lactobacillus brevis* reveals a significant plasmidome overlap of brewery and insect isolates. Curr. Microbiol..

[8] Koistinen V.M., Mattila O., Katina K., Poutanen K., Aura A.-M., Hanhineva K. (2018). Metabolic profiling of sourdough fermented wheat and rye bread. Sci. Rep..

[9] Heinl S., Grabherr R. (2017). Systems biology of robustness and flexibility: *Lactobacillus buchneri*-a show case. J. Biotechnol..

[10] Guo X.S., Ke W.C., Ding W.R., Ding L.M., Xu D.M., Wang W.W., Zhang P., Yang F.Y. (2018). Profiling of metabolome and bacterial community dynamics in ensiled *Medicago sativa* inoculated without or with *Lactobacillus plantarum* or *Lactobacillus buchneri*. Sci. Rep..

[11] Reale A., Di Renzo T., Rossi F., Zotta T., Iacumin L., Preziuso M., Parente E., Sorrentino E., Coppola R. (2015). Tolerance of *Lactobacillus casei*, *L. paracasei* and *L. rhamnosus* strains to stress factors encountered in food processing and in the gastro-intestinal tract. LWT Food Sci. Technol..

[12] Stefanovic E., Fitzgerald G., McAuliffe O. (2017). Advances in the genomics and metabolomics of dairy lactobacilli: A review. Food Microbiol..

[13] El Kafsi H., Binesse J., Loux V., Buratti J., Boudebbouze S., Dervyn R., Kennedy S., Galleron N., Quinquis B., Batto J.M. (2014). *Lactobacillus delbrueckii* ssp. *lactis* and ssp. *bulgaricus*: A chronicle of evolution in action. BMC Genom..

[14] Siezen R.J., Tzeneva V.A., Castioni A., Wels M., Phan H.T., Rademaker J.L., Starrenburg M.J., Kleerebezem M., Molenaar D., van Hylckama Vlieg J.E. (2010). Phenotypic and genomic diversity of strains isolated from various environmental niches. Environ. Microbiol..

[15] Mu Q., Tavella V.J., Luo X.M. (2018). Role of *Lactobacillus reuteri* in human health and diseases. Front. Microbiol..

[16] Capurso L. (2019). Thirty years of *Lactobacillus rhamnosus* GG: A review. J. Clin. Gastroenterol..

[17] Amadoro C., Rossi F., Piccirilli M., Colavita G. (2015). Features of *Lactobacillus sakei* isolated from Italian sausages: Focus on strains from Ventricina del Vastese. Ital. J. Food Saf..

[18] Eisenbach L., Geissler A.J., Ehrmann M.A., Vogel R.F. (2019). Comparative genomics of *Lactobacillus sakei* supports the development of starter strain combinations. Microbiol. Res..

[19] Gänzle M.G., Zheng J. (2018). Lifestyles of sourdough lactobacilli—Do they matter for microbial ecology and bread quality?. Int. J. Food Microbiol..

[20] Lee J.Y., Han G.G., Kim E.B., Choi Y.J. (2017). Comparative genomics of *Lactobacillus salivarius* strains focusing on their host adaptation. Microbiol. Res..

[21] Renouf V., Claisse O., Lonvaud-Funel A. (2005). Understanding the microbial ecosystem on the grape berry surface through numeration and identification of yeast and bacteria. Aust. J. Grape Wine Res..

[22] Franz C.M.A.P., Endo A., Abriouel H., Van Reenen C.A., Gálvez A., Dicks L.M., Holzapfel W.H., Wood B.J.B. (2014). The genus *Pediococcus*. Lactic Acid Bacteria: Biodiversity and Taxonomy.

[23] Amadoro C., Rossi F., Pallotta M.L., Gasperi M., Colavita G. (2018). Traditional dairy products can supply beneficial microorganisms able to survive in the gastrointestinal tract. LWT Food Sci. Technol..

[24] Dheeney D., Gareau M.G., Marco M.L. (2018). Intestinal *Lactobacillus* in health and disease, a driver or just along for the ride?. Curr. Opin. Biotechnol..

[25] Rossi M., Martinez-Martinez D., Amaretti A., Ulrici A., Raimondi S., Moya A. (2016). Mining metagenomic whole genome sequences revealed subdominant but constant *Lactobacillus* population in the human gut microbiota. Environ. Microbiol. Rep..

[26] Walter J. (2008). Ecological role of lactobacilli in the gastrointestinal tract: Implications for fundamental and biomedical research. Appl. Environ. Microbiol..

[27] Roos S., Engstrand L., Jonsson H. (2005). *Lactobacillus gastricus* sp. nov., *Lactobacillus antri* sp. nov., *Lactobacillus kalixensis* sp. nov. and *Lactobacillus ultunensis* sp. nov., isolated from human stomach mucosa. Int. J. Syst. Evol. Microbiol..

[28] Caufield P.W., Schön C.N., Saraithong P., Li Y., Argimón S. (2015). Oral lactobacilli and dental caries: A model for niche adaptation in humans. J. Dent. Res..

[29] Jespers V., Menten J., Smet H., Poradosú S., Abdellati S., Verhelst R., Hardy L., Buvé A., Crucitti T. (2012). Quantification of bacterial species of the vaginal microbiome in different groups of women, using nucleic acid amplification tests. BMC Microbiol..

[30] Nishiyama K., Sugiyama M., Mukai T. (2016). Adhesion properties of lactic acid bacteria on intestinal mucin. Microorganisms.

[31] Liu Y.W., Su Y.W., Ong W.K., Cheng T.H., Tsai Y.C. (2011). Oral administration of *Lactobacillus plantarum* K68 ameliorates DSS-induced ulcerative colitis in BALB/c mice via the anti-inflammatory and immunomodulatory activities. Int. Immunopharmacol..

[32] Wang Y., Wu Y., Wang Y., Xu H., Mei X., Yu D., Wang Y., Li W. (2017). Antioxidant properties of probiotic bacteria. Nutrients.

[33] Cohen P.A. (2019). Probiotic Safety—Reasonable Certainty of No Harm—Reply. JAMA Intern. Med..

[34] Boyle R.J., Robins-Browne R.M., Tang M.L.K. (2006). Probiotic use in clinical practice: What are the risks?. Am. J. Clin. Nutr..

[35] Sherid M., Samo S., Sulaiman S., Husein H., Sifuentes H., Sridhar S. (2016). Liver abscess and bacteremia caused by *Lactobacillus*: Role of probiotics? Case report and review of the literature. BMC Gastroenterol..

[36] Rocca M.F., Aguerre L., Cipolla L., Martínez C., Armitano R., Dangiolo G., Prieto M. (2018). *Lactobacillus* spp. invasive infections in Argentina. Int. J. Infect. Dis..

[37] Costa R.L., Moreira J., Lorenzo A., Lamas C.C. (2018). Infectious complications following probiotic ingestion: A potentially underestimated problem? A systematic review of reports and case series. BMC Complement. Altern. Med..

[38] Kamboj K., Vasquez A., Balada-Llasat J.M. (2015). Identification and significance of *Weissella* species infections. Front. Microbiol..

[39] Husni R.N., Gordon S.M., Washington J.A., Longworth D.L. (1997). *Lactobacillus* bacteremia and endocarditis: Review of 45 cases. Clin. Infect. Dis..

[40] Salminen M.K., Rautelin H., Tynkkynen S., Poussa T., Saxelin M., Valtonen V., Jarvinen A. (2004). *Lactobacillus* bacteremia, clinical significance, and patient outcome, with special focus on probiotic *L. rhamnosus* GG. Clin. Infect. Dis..

[41] Z’Graggen W.J., Fankhauser H., Lammer F., Bregenzer T., Conen D. (2005). Pancreatic necrosis infection due to *Lactobacillus paracasei* in an immunocompetent patient. Pancreatology.

[42] Cannon J.P., Lee T.A., Bolanos J.T., Danziger L.H. (2005). Pathogenic relevance of *Lactobacillus*: A retrospective review of over 200 cases. Eur. J. Clin. Microbiol. Infect. Dis..

[43] Horwitch C.A., Furseth H.A., Larson A.M., Jones T.L., Olliffe J.F., Spach D.H. (1995). Lactobacillemia in three patients with AIDS. Clin. Infect. Dis..

[44] Nishijima T., Teruya K., Yanase M., Tamori Y., Mezaki K., Oka S. (2012). Infectious endocarditis caused by *Lactobacillus acidophilus* in a patient with mistreated dental caries. Intern. Med..

[45] Brecht M., Garg A., Longstaff K., Cooper C., Andersen C. (2016). *Lactobacillus* sepsis following a laparotomy in a preterm infant: A note of caution. Neonatology.

[46] Ran L., Bégué R.E., Penn D. (2011). *Lactobacillus rhamnosus* sepsis in an infant without probiotic use: A case report and literature review. J. Neonat.-Perinat. Med..

[47] Land M.H., Rouster-Stevens K., Woods C.R., Cannon M.L., Cnota J., Shetty A.K. (2005). *Lactobacillus* sepsis associated with probiotic therapy. Pediatrics.

[48] Wagner R.D., Warner T., Roberts L., Farmer J., Balish E. (1997). Colonization of congenitally immunodeficient mice with probiotic bacteria. Infect. Immun..

[49] Gouriet F., Million M., Henri M., Fournier P.E., Raoult D. (2012). *Lactobacillus rhamnosus* bacteremia: An emerging clinical entity. Eur. J. Clin. Microbiol. Infect. Dis..

[50] Castro-González J.M., Castro P., Sandoval H., Castro-Sandoval D. (2019). Probiotic lactobacilli precautions. Front. Microbiol..

[51] Lockhart P.B., Brennan M.T., Sasser H.C., Fox P.C., Paster B.J., Bahrani-Mougeot F.K. (2008). Bacteremia associated with toothbrushing and dental extraction. Circulation.

[52] Lockhart P.B., Brennan M.T., Thornhill M., Michalowicz B.S., Noll J., Bahrani-Mougeot F.K., Sasser H.C. (2009). Poor oral hygiene as a risk factor for infective endocarditis-related bacteremia. J. Am. Dent. Assoc..

[53] Botros M., Mukundan D. (2014). *Lactobacillus* endocarditis with prosthetic material: A case report on non-surgical management with corresponding literature review. Infect. Dis. Rep..

[54] Aaron J.G., Sobieszczyk M.E., Weiner S.D., Whittier S., Lowy F.D. (2017). *Lactobacillus rhamnosus* endocarditis after upper endoscopy. Open Forum Infect. Dis..

[55] Boumis E., Capone A., Galati V., Venditti C., Petrosillo N. (2018). Probiotics and infective endocarditis in patients with hereditary hemorrhagic telangiectasia: A clinical case and a review of the literature. BMC Infect. Dis..

[56] Encarnacion C.O., Loranger A.M., Bharatkumar A.G., Almassi G.H. (2016). Bacterial endocarditis caused by *Lactobacillus acidophilus* leading to rupture of sinus of Valsalva aneurysm. Tex. Heart Inst. J..

[57] Groga-Bada P., Mueller I.I., Foschi F., Gawaz M., Eick C. (2018). Mitral valve endocarditis due to *Lactobacillus*. Case Rep. Med..

[58] Chen F., Zhang Z., Chen J. (2018). Infective endocarditis caused by *Lactococcus lactis* subsp. *lactis* and *Pediococcus pentosaceus*: A case report and literature review. Medicine (Baltimore).

[59] Oakey H.J., Harty D.W., Knox K.W. (1995). Enzyme production by lactobacilli and the potential link with infective endocarditis. J. Appl. Bacteriol..

[60] Cohen S.A., Woodfield M.C., Boyle N., Stednick Z., Boeckh M., Pergam S.A. (2016). Incidence and outcomes of bloodstream infections among hematopoietic cell transplant recipients from species commonly reported to be in over-the-counter probiotic formulations. Transpl. Infect. Dis..

[61] Haghighat L., Crum-Cianflone N.F. (2016). The potential risks of probiotics among HIV-infected persons: Bacteraemia due to *Lactobacillus acidophilus* and review of the literature. Int. J. STD AIDS.

[62] Holmberg P., Hellmich T., Homme J. (2017). Pediatric Sepsis Secondary to an occult dental abscess: A case report. J. Emerg. Med..

[63] Ambesh P., Stroud S., Franzova E., Gotesman J., Sharma K., Wolf L., Kamholz S. (2017). Recurrent *Lactobacillus* bacteremia in a patient with leukemia. J. Investig. Med. High Impact Case Rep..

[64] Datta P., Gupta V., Mohi G.K., Chander J., Janmeja A.K. (2017). *Lactobacillus coryniformis* causing pulmonary infection in a patient with metastatic small cell carcinoma: Case report and review of literature on *Lactobacillus* pleuro-pulmonary infections. J. Clin. Diagn Res..

[65] Biesiada G., Krycińska R., Czepiel J., Stażyk K., Kędzierska J., Garlicki A. (2019). Meningoencephalitis caused by *Lactobacillus plantarum*—Case report. Int. J. Neurosci..

[66] Darbro B.W., Petroelje B.K., Doern G.V. (2009). *Lactobacillus delbrueckii* as the cause of urinary tract infection. J. Clin. Microbiol..

[67] Duprey K.M., McCrea L., Rabinowitch B.L., Azad K.N. (2012). Pyelonephritis and bacteremia from *Lactobacillus delbrueckii*. Case Rep. Infect. Dis..

[68] Chazan B., Raz R., Shental Y., Sprecher H., Colodner R. (2008). Bacteremia and pyelonephritis caused by *Lactobacillus jensenii* in a patient with urolithiasis. Isr. Med. Assoc. J..

[69] Citla S.D., Gourishankar A. (2013). *Lactobacillus* causing urinary tract infection in a neonate. J. Med. Cases.

[70] Kirjavainen P.V., Tuomola E.M., Crittenden R.G., Ouwehand A.C., Harty D.W., Morris L.F., Rautelin H., Playne M.J., Donohue D.C., Salminen S.J. (1999). In vitro adhesion and platelet aggregation properties of bacteremia-associated lactobacilli. Infect. Immun..

[71] Nissilä E., Douillard F.P., Ritari J., Paulin L., Järvinen H.M., Rasinkangas P., Haapasalo K., Meri S., Jarva H., de Vos W.M. (2017). Genotypic and phenotypic diversity of *Lactobacillus rhamnosus* clinical isolates, their comparison with strain GG and their recognition by complement system. PLoS ONE.

[72] Fitzgerald J.R., Foster T.J., Cox D. (2006). The interaction of bacterial pathogens with platelets. Nat. Rev. Microbiol..

[73] Collins J., van Pijkeren J.P., Svensson L., Claesson M.J., Sturme M., Li Y., Cooney J.C., van Sinderen D., Walker A.W., Parkhill J. (2012). Fibrinogen-binding and platelet-aggregation activities of a *L. salivarius* septicaemia isolate are mediated by a novel fibrinogen-binding protein. Mol. Microbiol..

[74] Abriouel H., Lerma L.L., Casado Muñoz Mdel C., Montoro B.P., Kabisch J., Pichner R., Cho G.S., Neve H., Fusco V., Franz C.M. (2015). The controversial nature of the *Weissella* genus: Technological and functional aspects versus whole genome analysis-based pathogenic potential for their application in food and health. Front. Microbiol..

[75] Floch M.H. (2018). The role of prebiotics and probiotics in gastrointestinal disease. Gastroenterol. Clin. N. Am..

[76] Cai J., Zhao C., Du Y., Zhang Y., Zhao M., Zhao Q. (2018). Comparative efficacy and tolerability of probiotics for antibiotic-associated diarrhea: Systematic review with network meta-analysis. United Eur. Gastroenterol. J..

[77] Gorbach S., Doron S., Magro F., Floch M., Ringel Y., Walker W.A. (2016). Chapter 7—Lactobacillus rhamnosus GG. The Microbiota in Gastrointestinal Pathophysiology. Implications for Human Health, Prebiotics, Probiotics, and Dysbiosis.

[78] Mantegazza C., Molinari P., D’Auria E., Sonnino M., Morelli L., Zuccotti G.V. (2018). Probiotics and antibiotic-associated diarrhea in children: A review and new evidence on *Lactobacillus rhamnosus* GG during and after antibiotic treatment. Pharmacol. Res..

[79] Tytgat H.L., Douillard F.P., Reunanen J., Rasinkangas P., Hendrickx A.P., Laine P.K., Paulin L., Satokari R., de Vos W.M. (2016). *Lactobacillus rhamnosus* GG outcompetes *Enterococcus faecium* via mucus-binding pili: Evidence for a novel and heterospecific probiotic mechanism. Appl. Environ. Microbiol..

[80] Petrova M.I., Imholz N.C., Verhoeven T.L., Balzarini J., Van Damme E.J., Schols D., Vanderleyden J., Lebeer S. (2016). Lectin-like molecules of *Lactobacillus rhamnosus* GG inhibit pathogenic *Escherichia coli* and *Salmonella* biofilm formation. PLoS ONE.

[81] Wuyts S., Wittouck S., De Boeck I., Allonsius C.N., Pasolli E., Segata N., Lebeer S. (2017). Large-scale phylogenomics of the *Lactobacillus casei* group highlights taxonomic inconsistencies and reveals novel clade-associated features. MSystems.

[82] Costabile A., Bergillos-Meca T., Rasinkangas P., Korpela K., de Vos W.M., Gibson G.R. (2017). Effects of soluble corn fiber alone or in synbiotic combination with *Lactobacillus rhamnosus* GG and the pilus-deficient derivative GG-PB12 on fecal microbiota, metabolism, and markers of immune function: A randomized, double-blind, placebo-controlled, crossover study in healthy elderly (Saimes study). Front. Immunol..

[83] Pace F., Pace M., Quartarone G. (2015). Probiotics in digestive diseases: Focus on *Lactobacillus* GG.. Miner. Gastroenterol. Dietol..

[84] Blaabjerg S., Artzi D.M., Aabenhus R. (2017). Probiotics for the prevention of antibiotic-associated diarrhea in outpatients-a systematic review and meta-analysis. Antibiotics (Basel).

[85] Pessi T., Sütas Y., Hurme M., Isolauri E. (2000). Interleukin-10 generation in atopic children following oral *Lactobacillus rhamnosus* GG. Clin. Exp. Allergy.

[86] Viljanen M., Savilahti E., Haahtela T., Juntunen-Backman K., Korpela R., Poussa T., Tuure T., Kuitunen M. (2005). Probiotics in the treatment of atopic eczema/dermatitis syndrome in infants: A double-blind placebo-controlled trial. Allergy.

[87] Laursen R.P., Hojsak I. (2018). Probiotics for respiratory tract infections in children attending day care centers-a systematic review. Eur. J. Pediatr..

[88] Rungsri P., Akkarachaneeyakorn N., Wongsuwanlert M., Piwat S., Nantarakchaikul P., Teanpaisan R. (2017). Effect of fermented milk containing *Lactobacillus rhamnosus* SD11 on oral microbiota of healthy volunteers: A randomized clinical trial. J. Dairy Sci..

[89] Lee K., Lee Y. (2009). Production of c9,t11- and t10,c12-conjugated linoleic acids in humans by *Lactobacillus rhamnosus* PL60. J. Microbiol. Biotechnol..

[90] Braat H., van den Brande J., van Tol E., Hommes D., Peppelenbosch M., van Deventer S. (2004). *Lactobacillus rhamnosus* induces peripheral hyporesponsiveness in stimulated CD4+ T cells via modulation of dendritic cell function. Am. J. Clin. Nutr..

[91] Gill H.S., Rutherfurd K.J., Cross M.L. (2001). Dietary probiotic supplementation enhances natural killer cell activity in the elderly: An investigation of age-related immunological changes. J. Clin. Immunol..

[92] Salminen M.K., Rautelin H., Tynkkynen S., Poussa T., Saxelin M., Valtonen V., Järvinen A. (2006). *Lactobacillus* bacteremia, species identification, and antimicrobial susceptibility of 85 blood isolates. Clin. Infect. Dis..

[93] Meini S., Laureano R., Fani L., Tascini C., Galano A., Antonelli A., Rossolini G.M. (2015). Breakthrough of *Lactobacillus rhamnosus* GG bacteremia associated with probiotic use in an adult patient with severe active ulcerative colitis: Case report and review of the literature. Infection.

[94] Kulkarni H.S., Khoury C.C. (2014). Sepsis associated with *Lactobacillus* bacteremia in a patient with ischemic colitis. Indian J. Crit. Care Med..

[95] Dani C., Coviello C., Corsini I., Arena F., Antonelli A., Rossolini G.M. (2016). *Lactobacillus* Sepsis and probiotic therapy in newborns: Two new cases and literature review. AJP Rep..

[96] Luong M.-L., Sareyyupoglu B., Nguyen M.H., Silveira F.P., Shields R.K., Potoski B.A., Pasculle W.A., Clancy C.J., Toyoda Y. (2010). *Lactobacillus* probiotic use in cardiothoracic transplant recipients: A link to invasive *Lactobacillus* infection?. Transpl. Infect. Dis..

[97] Doern C.D., Nguyen S.T., Afolabi F., Burnham C.A. (2014). Probiotic-associated aspiration pneumonia due to *Lactobacillus rhamnosus*. J. Clin. Microbiol..

[98] Sadowska-Krawczenko I., Paprzycka M., Korbal P., Wiatrzyk A., Krysztopa-Grzybowska K., Polak M., Czajka U., Lutyńska A. (2014). *Lactobacillus rhamnosus* GG suspected infection in a newborn with intrauterine growth restriction. Benef. Microb..

[99] Koyama S., Fujita H., Shimosato T., Kamijo A., Ishiyama Y., Yamamoto E., Ishii Y., Hattori Y., Hagihara M., Yamazaki E. (2018). Septicemia from *Lactobacillus rhamnosus* GG, from a probiotic enriched yogurt, in a patient with autologous stem cell transplantation. Probiotics Antimicrob. Proteins.

[100] Alander M., Satokari R., Korpela R., Saxelin M., Vilpponen-Salmela T., Mattila-Sandholm T., von Wright A. (1999). Persistence of colonization of human colonic mucosa by a probiotic strain, *Lactobacillus rhamnosus* GG, after oral consumption. Appl. Environ. Microbiol..

[101] Tynkkynen S., Satokari R., Saarela M., Mattila-Sandholm T., Saxelin M. (1999). Comparison of ribotyping, randomly amplified polymorphic DNA analysis, and pulsed-field gel electrophoresis in typing of *Lactobacillus rhamnosus* and *L. casei* strains. Appl. Environ. Microbiol..

[102] Ceapa C., Lambert J., van Limpt K., Wels M., Smokvina T., Knol J., Kleerebezem M. (2015). Correlation of *Lactobacillus rhamnosus* genotypes and carbohydrate utilization signatures determined by phenotype profiling. Appl. Environ. Microbiol..

[103] Nadkarni M.A., Chen Z., Wilkins M.R., Hunter N. (2014). Comparative genome analysis of *Lactobacillus rhamnosus* clinical isolates from initial stages of dental pulp infection: Identification of a new exopolysaccharide cluster. PLoS ONE.

[104] Rossi F., Zotta T., Iacumin L., Reale A. (2016). Theoretical insight into the heat shock response (HSR) regulation in *Lactobacillus casei* and *L. rhamnosus*. J. Theor. Biol..

